# A Quantum Mechanism-Centered Review of Carbon Dot-Based Theranostics

**DOI:** 10.3390/ijms27209016

**Published:** 2026-10-12

**Authors:** Ian Ke Cheng, Gregory P. Contreras, Ting-Yu Yeh

**Affiliations:** 1 Auxergen Inc., Rita Rossi Colwell Center, 701 E Pratt Street, Room 3017, Baltimore, MD 21202, USAgreg@auxergen.com (G.P.C.); 2 Auxergen S.r.l., Tecnopolis PST, University of Bari, Valenzano, 70010 Bari, Italy

**Keywords:** carbon dots (CDs), quantum mechanism, drug delivery, photodynamic therapy

## Abstract

Despite advances in drug discovery, effective delivery and precise imaging still pose significant obstacles. Scientists have turned to zero-dimensional nanomaterials like carbon dots (CDs) to enable precision drug delivery. Due to their multidisciplinary appeal, there is extensive research on both the physical properties and biological applications of CDs, yet the fields rarely complement each other. This review applies a quantum-mechanistic framework to better understand CDs and unify the relationship between electronic structure, photophysical behavior, and theranostic performance. We first highlight the theranostic advantages of CDs over quantum dots, then discuss the quantum mechanisms behind a CD’s imaging capabilities, including surface states, excitation-dependent photoluminescence, and the electronic origins of photostability. Through this physical lens, we then evaluate the effect of different precursors and doping strategies during synthesis on electronic structure and how dots are engineered to load, release, and track therapeutic cargo. Photodynamic therapy and surface functionalization strategies are also evaluated. Finally, we address the significance of renal clearance and translational bottlenecks such as heterogeneity.

## 1. Introduction

One of the most pressing, multifaceted issues in healthcare is the inefficiency of the drug development process: 90% of drug candidates fail. In their use of the structure–activity relationship, the pharma and biotech industries largely focus on how potent drugs are against specific targets. During drug optimization, there is less consideration of the structure–tissue exposure/selectivity relationship. Current algorithms often fail to realize just how big a role structural properties like molecular size and permeability play in determining the drug’s accumulation and selectivity in prospective biological targets [1]. For example, when it comes to the blood–brain barrier (BBB), a lack of knowledge of the transport properties of its endothelial cells is why the BBB hampers growth in neurotherapeutics [2].

Another cause of the problem is toxicity, which accounts for about one-third of drug development failures. Some cancer drugs target proteins that do not contribute to cancer cell proliferation, potentially leading to adverse effects [3]. Yet, toxicity is often recognized late in trials, making early detection imperative [4].

The solution lies in theranostics, which, in the context of nanomedicine, synchronizes deep-tissue tracking with localized therapeutic action [5]. In vitro, the imaging and delivery purposes have already benefited from the implementation of quantum dots (QDs), a zero-dimensional (0D) fluorescence-type nanocrystal.

A QD has a semiconductor core composed of either cadmium sulfide, cadmium selenide, or cadmium telluride. An outer shell insulates the sensitive core from elements like oxygen, preventing photo-oxidation. In addition, the shell’s material has a wider bandgap than that of the semiconductor, providing a barrier that prevents charge carriers from leaving the core. The result is increased quantum yield [6,7]. This structure makes the QD an efficient fluorescent label in a drug delivery system. A QD is also relatively small compared to a cell membrane (7–10 nm thick), allowing it to enter cells via fluid-phase endocytosis [8]. Even so, their heavy, inorganic core makes their use in drug delivery dangerous. An emerging alternative is the carbon dot (CD). Ranging from 1.8 to 10 nm in size, it possesses similar quantum properties to the QD [9]. However, the key differences lie in the following metrics: toxicity, solubility, and chemical versatility.

### 1.1. Toxicity

In both nanomaterials, toxicity is determined by the material of the outermost layer. As we know, QDs have an inorganic, metallic core whose toxicity is reduced by the outer shell. In contrast, the core structure of a CD is composed of a mixture of graphitic (sp^2^) and disordered (sp^3^) carbon atoms. This gives it the advantage of being chemically inert. Even if a CD breaks down in the body, its naturally occurring material makes it harmless [10,11,12].

### 1.2. Solubility

If a drug is water-insoluble, a higher dose needs to be administered for it to reach therapeutic pharmaceutical concentrations. Consequently, soluble drugs have better bioavailability [13]. Since most QD cores are hydrophobic, their surface needs to be capped with hydrophilic ligands before they can deliver soluble drugs. This can be a difficult and expensive process [10,14]. In contrast, the surface functional groups and the hydrophilic core of a CD both enhance its intrinsic aqueous solubility, which is why it is generally deemed better for therapy delivery [15].

### 1.3. Chemical Versatility/Biocompatibility

The agents on a QD or CD’s outermost surface determine its final application [16]. Structurally, CDs have an abundance of functional groups (-OH, -COOH, NH2), allowing for more complex bioconjugation. They also readily respond to environmental stimuli such as pH and enzymatic activity. Their surface can also be oxidized to adjust fluorescence, improving their diagnostic ability. On the other hand, a QD only offers two functional groups (-COOH and -NH2) for binding with drug molecules. It is more difficult for the environment to automatically trigger a drug’s release [9,10].

**Figure 1:**
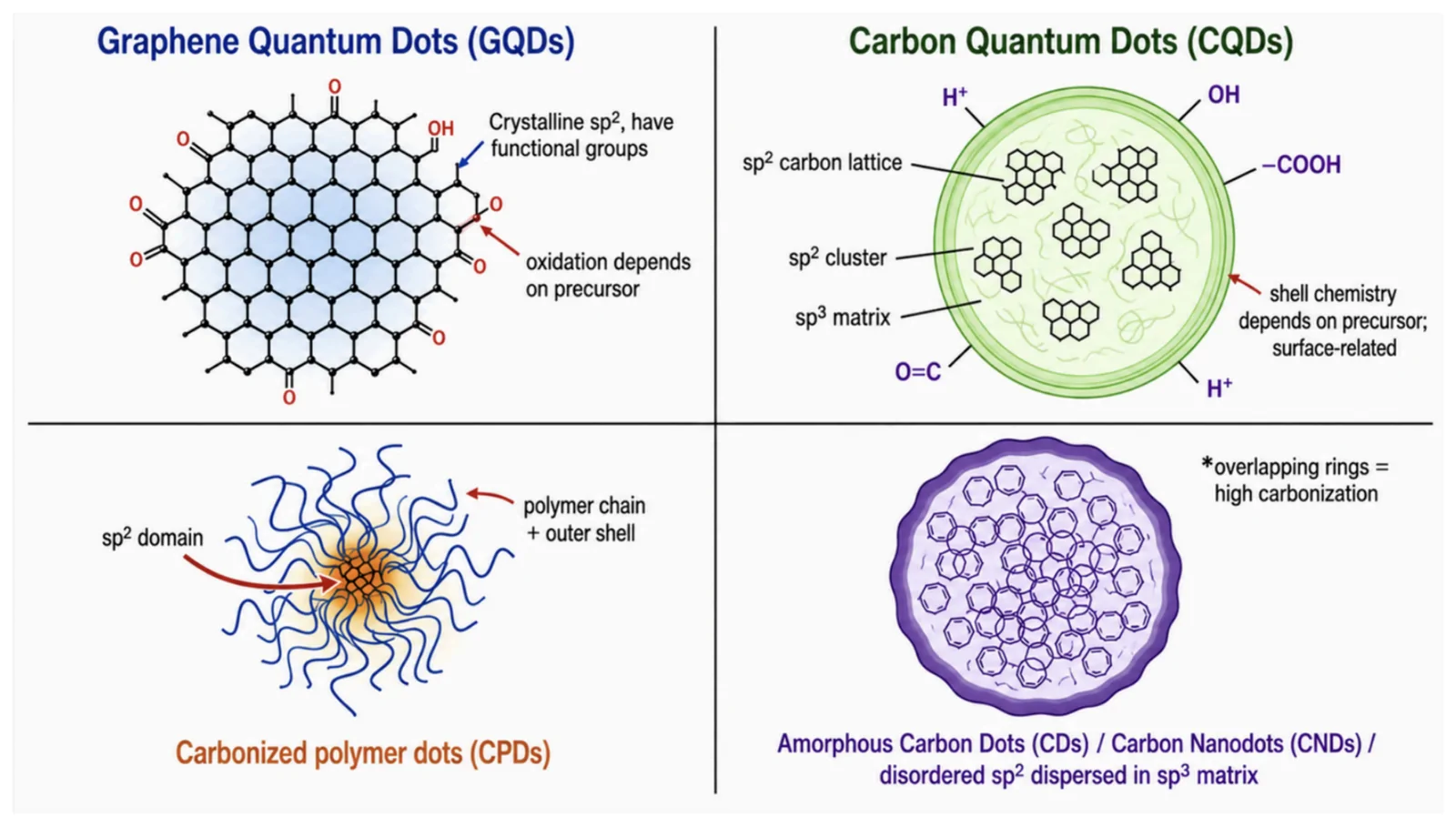
Diagram comparing graphene quantum dots (GQDs), carbon quantum dots (CQDs), and carbon polymer dots (CPDs). * Overlapping rings represent high carbonization.

Generally, CDs are categorized into graphene quantum dots (GQDs), carbon quantum dots (CQDs), and carbonized polymer dots (CPDs). A key distinction between the three groups is the carbon source. GQDs are usually synthesized via top-down methods. As a result, they retain a graphene structure. This consists of two nested triangular sublattices that interlock to form crystalline six-membered ring structures, where carbon atoms are connected to three other carbon neighbors by sigma bonds resulting from sp^2^ hybridization. There is no sp^3^ carbon insulator; therefore, quantum confinement is limited. On the other hand, bottom-up methods are used to synthesize CQDs from a variety of carbon sources, including biomass, carbohydrates, and organic waste. While the core of a CQD is also graphitic and made of tightly packed C–C bonds, these sp^2^ clusters are isolated within a surrounding sp^3^ matrix. This confines the delocalized pi electrons to a greater extent than in GQDs, creating more localized luminescent centers and increasing quantum yield. In the scientific literature and in this review, the term CQD is used interchangeably with the term CD [11,17,18]. The structural differences between the CDs are explained in Figure 1. Moreover, the structural differences illustrate the quantum-mechanistic framework of this review. Quantum/electronic and photophysical mechanisms, such as quantum confinement, surface states, charge transfer, intersystem crossing, and spin–orbit coupling, are distinct from downstream chemical, interfacial, and biological mechanisms like drug release, but their causal connections are discussed.

To address these limitations, this paper applies a quantum-mechanistic framework to connect the structural physics of carbon dots to their application in theranostic systems. The following sections discuss the photophysics behind bioimaging (Section 2), how these properties are tuned through synthesis (Section 3), drug loading, stimuli-responsive release, and precision drug delivery (Section 4), and the translational barriers to human clinical trials (Section 5).

## 2. Quantum Mechanics of Imaging

The clinical application of 0D carbon materials could be better understood through their unique physics. Thus, this section describes the quantum mechanisms driving the precise imaging capabilities of CDs.

### 2.1. Quantum Confinement

The 2–10 nm size of a CD limits electron motion to the point that it can no longer possess a continuous spectrum of energy, causing energy levels to become discrete. This is known as the quantum confinement effect, which means that its optical and electrical properties can be tuned by simply changing the dot’s size. For example, during band gap absorption, electrons will only absorb levels of energy high enough to reach the conduction band. If there is too much energy, the electron will relax until it reaches the exact energy band. As a result, when falling to the valence band, it emits a photon of an exact energy, allowing for precise colors [19].

This is validated through Park et al.’s synthesis of luminescent CQDs using paper substrates and heat treatment. Ultraviolet–visible absorption spectroscopy revealed an absorbance peak at 264 nm and an emission peak at about 430 nm (blue light). Moreover, this difference between absorption and emission wavelengths, known as the Stokes Shift, allows microscope filters to separate the absorbed light from the emitted light, in turn creating clear bioimages [20,21].

### 2.2. Excitation-Dependent Emission and Surface States

A single CD has the capacity to emit multiple different colors owing to the quantum property of excitation-dependent emission. This process is driven by the fact that CD samples are very rarely identical: varying surface functional groups introduce multiple varying surface states, or localized energy levels that exist within the dot’s bandgap [22].

**Figure 2:**
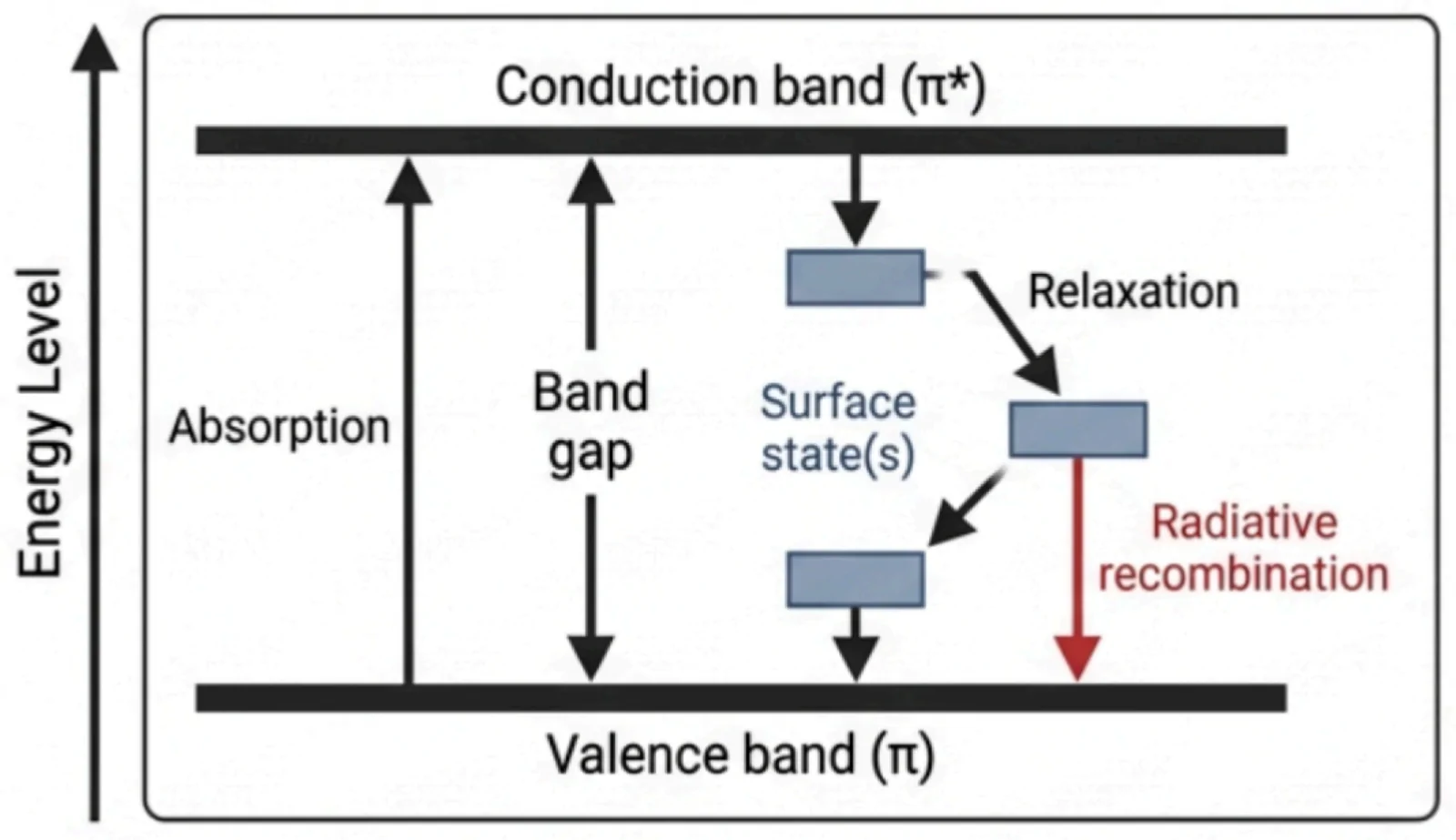
Band alignment and excitation-dependent emission (EDE): conduction/valence band energy levels illustrating EDE originating from surface trapping vs. core states.

Surface states are “synergistic hybridizations”, meaning that the carbon of the inner core and the functional groups on the shell chemically merge into a disorderly and heterogeneous network. For example, sp^3^-hybridized carbons create traps that could interrupt the flow of electrons across the sp^2^-carbon network. In this situation, after falling from the conduction band and then relaxing in the traps (releasing heat), the electrons will fall back down into the valence band. They then release a different color of light than they would from a direct drop from the conduction to the valence band. This property is useful because it makes CDs photoselectable. In other words, a specific wavelength of light will only excite a specific sub-population of surface states that can absorb said wavelength. Thus, only this sub-population undergoes radiative recombination, allowing scientists to control the color of emitted light [23,24]. This fluorescence mechanism is why CD photoluminescence is also said to be surface state-dictated rather than size-dictated as the quantum confinement effect suggests. This mechanism is visualized in Figure 2.

It is also important to consider that surface states created by excessive doping or uncontrolled carbonization can be non-radiative, meaning exciton energy is completely lost to heat. This is an ongoing challenge toward maximizing CD fluorescence efficiency. However, non-radiative recombination can also be a desirable therapeutic effect, as it can result in heat generation and ROS production. Photodynamic therapy, a ROS-based therapeutic mechanism, is explained separately in Section 4 [25]. This demonstrates an example of how imaging and therapy can be competing outcomes while designing CD theranostic systems.

### 2.3. Near-Infrared (NIR) Emission

NIR light is characterized by its relatively long wavelengths (700–1700 nm), putting it in the “biological optical window”. Leveraging this is often discussed in modern-day nanomedicine, as NIR-responsive CDs yield a high signal-to-background ratio. This prevents the background noise caused by cell and tissue autofluorescence and makes it much easier to track dot location and transfer energy to it in vivo [26,27].

The diagnostic advantages of NIR have already been tested. In a 2016 study, Jeong et al. created QDs with a lead sulfide (PbS) core, cadmium sulfide (CdS) shell, and zinc sulfide (ZnS) shell, giving them the ability to emit NIR-II (1000–1700 nm) light and a quasi-type II band alignment [28]. In this state, the shell’s conduction band is aligned almost perfectly with the core’s conduction band. In turn, excited electrons are able to escape to the shell’s conduction band. They no longer have to recombine with their hole and can instead interact with the quencher [29].

Despite their imaging potential, the issue with using these dots clinically is toxicity from heavy-metal (Pb, Cd) leaching. More recent CDs have both overcome this limitation and maintained equivalent optical performance. Through pyrrolic nitrogen doping, Yang et al. engineered a surface state near the valence band, prompting excitons to release a low-energy NIR-II wave [30].

### 2.4. Up-Conversion Fluorescence

This property is defined as the absorption of two or more low-energy photons to emit one higher-energy photon, classifying it as an anti-Stokes process. The presence of stable intermediate energy levels allows for the slower, sequential buildup of photons before release [31]. In in vitro bioimaging, this is useful because CDs can convert multiple deep-penetrating NIR waves into a single higher-energy light wave, improving imaging contrast and spatial clarity.

Up-conversion fluorescence is related to, yet distinct from, multiphoton microscopy, where two photons must excite a molecule almost simultaneously for the same effect. When the first photon hits, the molecule will be pushed up into a virtual energy state [32].

**Figure 3:**
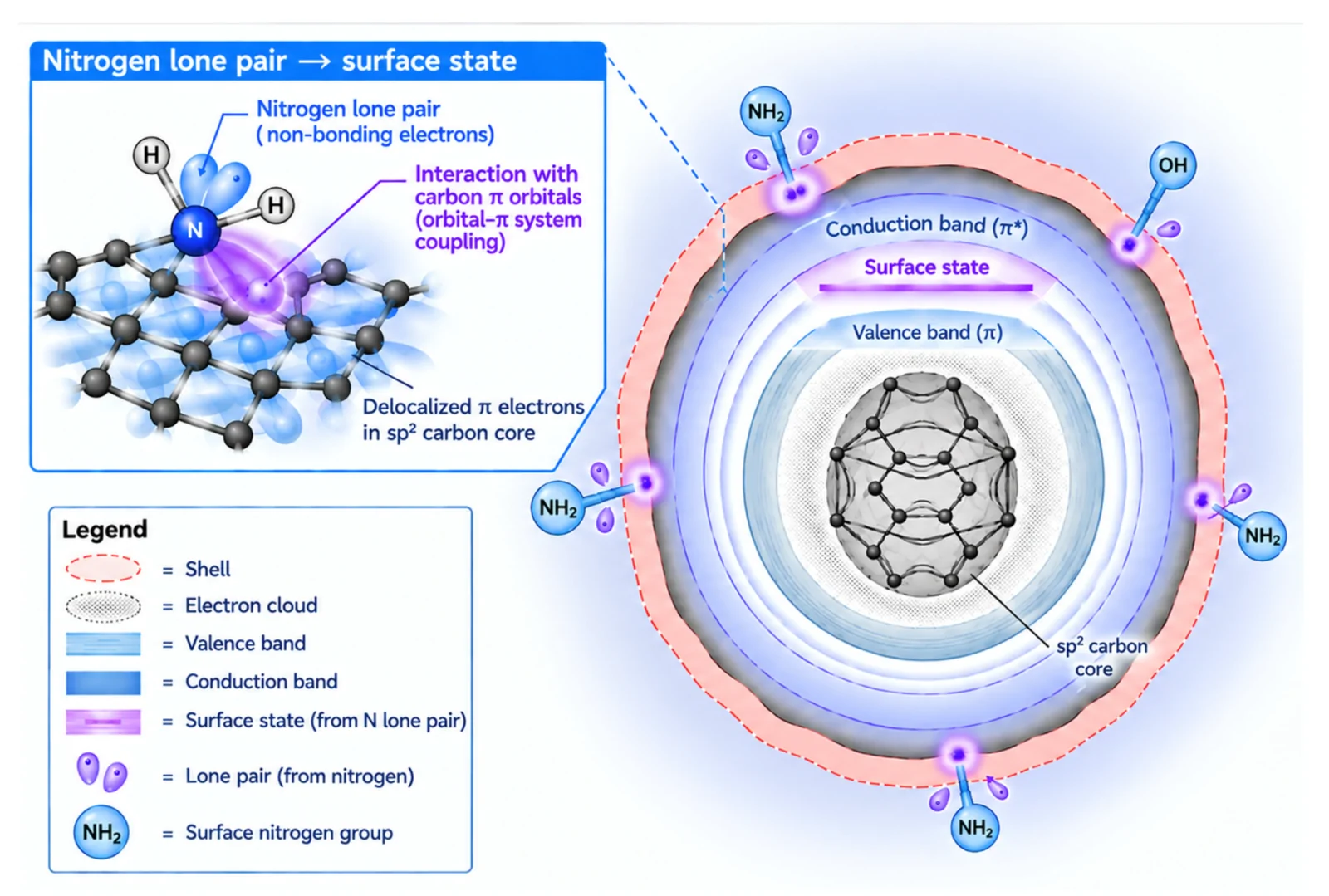
Structure and cloud: sp^2^/sp^3^ carbon core showing the delocalized pi-electron cloud and surface oxidation states. Bright purple spots near the shell represent surface states. π* conduction band is an energy band formed by unoccupied, antibonding π molecular orbitals.

Both mechanisms are driven by two-photon absorption, which is demonstrated in Lesani et al.’s study. Their CDs possessed large, flat, sp^2^ carbon domains, forming a delocalized pi-electron cloud. This cloud is highly polarizable, increasing the CD’s two-photon absorption cross-section and thus making it easier for the dot to absorb two photons simultaneously [33]. The creation of an electron cloud is visualized in Figure 3.

### 2.5. Photostability

Organic dyes are commonly used to monitor chronic conditions. For instance, when highly diluted and safely sprayed on the colon epithelium, methylene blue is useful as it creates a clear visual contrast between healthy cells and polyps, helping in cancer detection [34].

The problem is that organic dyes, many of which are also fluorophores, photobleach easily [35]. After absorbing a photon, a dye molecule is boosted to a singlet state. From there, it is very reactive and, via intersystem crossing, can enter a triplet state. In a singlet state, the spin of an electron and its corresponding hole are in opposite directions, allowing the electron to fall back down to the ground state. Conversely, in a triplet state, the excited electron flips its spin so both it and the hole are spinning in the same direction. The rules of quantum mechanics dictate that it cannot fall back into the hole, so it is temporarily trapped in that higher-energy state. This not only prevents light emission, but also allows the electron to interact with its surroundings, such as ambient oxygen (O2). The electron’s transfer of energy to the oxygen can sensitize the creation of reactive singlet oxygen, a highly destructive reactive oxygen species (ROS). After ISC, ROS readily cleave covalent bonds, irreversibly oxidizing and damaging the dye’s chemical structure and permanently preventing fluorescence [35,36,37].

In contrast, CQDs have remarkably high photostability. When exposed to a UV lamp (365 nm, 5 W, 20 cm away) for 264 h, NaCl salt crystal-embedded CDs stabilized at around the 200th hour and retained over 70% of their initial photoluminescence intensity level. This 30–40% degradation was not attributed to light, but to the water and oxygen trapped in the salt crystals during synthesis. Theoretically, it should respond to light indefinitely if synthesized carefully and properly [38].

**Figure 4:**
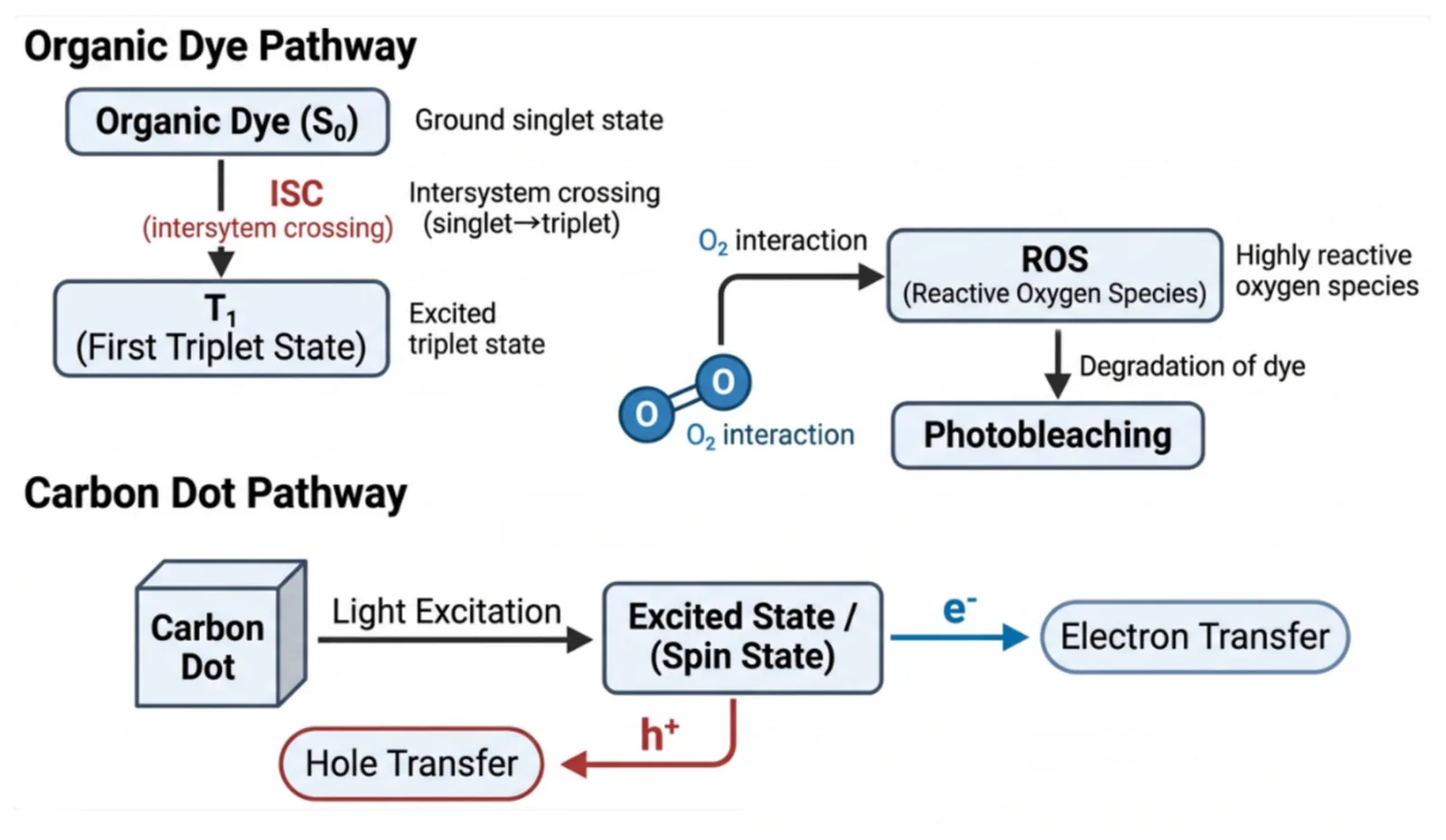
Spin dynamics and photostability: diagram showing how sp^2^ domain confinement suppresses intersystem crossing (ISC) to prevent photobleaching.

Moreover, the rigidly conjugated sp^2^ carbon sheet of a CD distributes exciton energy efficiently through Förster-type resonance energy transfer between localized sp^2^ domains and surface states [39,40]. This mechanism was validated in N,S co-doped CQDs, revealing multiexponential carrier relaxation ranging from 0.5 picoseconds to >500 picoseconds [41]. While doping with heavy atoms or heteroatoms facilitates intersystem crossing (ISC), pure CDs (e.g., GQDs) exhibit slow ISC kinetics, taking approximately 10–20 nanoseconds to reach the triplet state [42,43]. Since electron energy is quickly dissipated before ISC can occur, the chance that a carbon dot will enter a triplet state and photobleach is minimized. Doping and its effects on ISC are further discussed in Section 3. The differences between organic dyes and CD excitation are explained in Figure 4.

Yet, a limitation is that synthesis of CDs can leave behind impurities, which can reach the triplet state and photobleach. Thus, CDs must undergo rigorous purification to ensure stability, which can hinder their appeal as a theranostic agent [44].

## 3. Synthesis

The methods to synthesize nanomaterials are categorized into top-down and bottom-up. Top-down methods generally involve the division of bulk materials into nanoparticles. It is less favored for CD synthesis because scientists have less control over its final properties [45]. In contrast, in bottom-up processes like green synthesis, secondary metabolites and active proteins determine the synthesized nanomaterial’s structural properties, and in turn, its function [46,47].

For example, CDs synthesized from the leaves of *Azadirachta indica*, an indigenous medicinal plant known as neem or the Indian lilac, are powerful antioxidants and inhibitors of bacteria and fungi. *Azadirachta indica* contains various polyphenolic compounds like gallic acid, which possess high antioxidant activity. This is because the hydroxyl groups in the compound act as electron donors, neutralizing free radicals before they can damage cellular structures. Consequently, the synthesized CDs will carry similar, if not the same, chemical functional groups, therefore inheriting the plant’s properties [48,49]. While neem-derived CDs are intrinsically fluorescent, optimization of their quantum yield is necessary to expand their efficacy in advanced optical and bioimaging applications. This can be performed by changing the carbon precursor, which affects both nanomaterial function and electronic structure. The mechanism relies on tuning the bandgap through the fraction of sp^2^-hybridized carbon atoms embedded inside an sp^3^ carbon atom matrix. Inside a CD’s core, the surrounding sp^3^ network acts as an insulator, confining electron–hole pairs within the sp^2^ clusters, which turns those clusters into luminescent centers. Using different carbon precursors changes this ratio, altering energy levels and, subsequently, photoluminescence [11,50,51]. Lesani et al.’s CDs (discussed in Up-Conversion Fluorescence) were synthesized with aromatic precursors, which contain highly stable sp^2^ carbon rings. The fusion of these rings extends the conjugated domain, allowing electrons to delocalize over a greater area, which then compresses the gap between the valence and conduction bands (also known as the HOMO–LUMO gap). Photoluminescence was shifted into the low-energy NIR region as a result [33].

Another strategy to optimize quantum yield is doping, a process where scientists intentionally insert heteroatoms, either during or after synthesis, to fine-tune a CQD’s properties. However, doping is not limited to CQDs. In reality, modern-day CDs are usually structural hybrids of CQDs, GQDs, and CPDs, rather than strictly one type of dot. For example, the core of a CPD is a highly cross-linked polymer network with a low level of carbonization. This is because some precursors cannot be carbonized completely during bottom-up synthesis, resulting in both CQD and CPD-like properties. Consequently, the incorporation of dopants alters a dot’s electronic structure across the entire material spectrum [52,53]. For example, nitrogen doping increased photoluminescence quantum yield by 8–9%. The element’s addition introduced surface passivation and tuned the energy gap width. The impact of these modifications is reflected in the surface charge of the nanoparticles. When palm empty fruit bunches, the leftover woody stalk left over during the palm oil milling process, were used as a raw precursor to synthesize a CD, the resulting zeta potential was −14.6 mV. However, when doped with nitrogen-containing urea, this value was partially balanced by the positive charges introduced by amines (NH2) (−13.10 to −10.27 mV). In contrast, sulfate doping shifts the charge to be more negative (−18.2 to −18.5 mV), maximizing negative electrostatic repulsion and thereby ensuring colloidal stability in aqueous environments [54,55].

In Yang et al.’s study (discussed in Near-Infrared Emission), pyrrolic nitrogen doping was specifically effective in creating a surface state. Its higher electronegativity relative to carbon breaks structural symmetry and creates a localized region, as indicated by an increased molecular dipole moment [30]. Pyrrolic nitrogen then donates its lone electron (Pz) pair to the conjugated pi-system. This localized increase in electron density forces the formation of a new surface state, as the excess charge now cannot fit into the valence band. Notably, nitrogen doping worked in tandem with an extended sp^2^ domain to shrink the energy gap, demonstrating both the adaptability and versatility of bottom-up carbon dot synthesis [30].

Another critical effect of doping is its potential to accelerate ISC kinetics. While this is unfavorable for imaging purposes, ISC is necessary for therapeutic processes, especially photodynamic therapy (PDT, discussed in Section 4). Heteroatoms are a source of non-bonding (n) lone-pair electrons, enabling orbital character change (1(–*) → 3(n–*)). According to El-Sayed’s Rule, ISC under this condition occurs orders of magnitude faster than without orbital character change (1(–*) → 3(–*)), which is the case in pristine CDs. Therefore, electrons quickly enter the triplet state, facilitating the creation of ROS. This increase is also amplified by the heavy-atom effect: sulfur doping (Z = 16), due to having a larger atomic number than nitrogen (Z = 7), exerts stronger spin–orbit coupling (the magnetic interaction between an electron’s orbital motion and its intrinsic spin that enables spin flips) and results in a faster ISC rate [42,56].

This chemical versatility ensures that the surface of CDs can be functionalized not only for bioimaging, but also for theranostic purposes. This is important to move away from simple passive targeting, such as reliance on the enhanced permeability and retention effect. CDs can be engineered for active targeting, making them better positioned to bypass physiological barriers. Due to the functional groups on a CD’s shell, ligands that interact with the target can be attached to its surface. Said ligands can either be chemically conjugated to functional groups on the nanoparticle or integrated with nanoparticle components pre-synthesis. The latter is also called in situ doping [57,58].

## 4. Precision Drug Delivery

The various examples cited in this paragraph are summarized in Table 1.

### 4.1. Loading Strategies

One way drugs are conjugated to CDs is through covalent bonds, such as amide, ester, and hydrazine links. Each has its own advantage: ester links can be broken down by enzymes for controlled release, and hydrazine links cleave in acidic environments [59]. Another method is physical encapsulation, where the drug is incorporated within the CD’s framework through weaker non-covalent forces. While it can be performed post-synthesis in a relatively simple way, giving it adaptability, covalent bonding is usually favored for its lowered risk of early, unintended leakage [60].

When Mohammed et al. synthesized nitrogen-doped CDs, the flat aromatic benzene rings aligned and stacked on each other, constructing the carbon core via stacking. Post-synthesis, this provides a platform for anticancer drugs, many of which also possess aromatic rings, to be loaded. The electron clouds of the drug molecules and the CD surface align, allowing for loading right on the CD’s graphitic surface. When these interactions occur, absorbance peaks increase. The merged electron clouds expand the surface areas over which electrons can roam and introduce new surface states, shrinking the energy gap/band gap [61,62,63].

### 4.2. Stimuli-Responsive Release

#### 4.2.1. pH Changes

Drug delivery systems discussed in this category release cargo only in acidic environments, namely tumors. For example, Ahamed et al. attached the anticancer peptide Proximicin-A to a CD through an imine bond (C=N). Protonation in an acidic tumor environment triggered hydrolysis and, in turn, drug release. Further analysis revealed that the energy gap/HOMO–LUMO gap shrank in acidic conditions. The conjugated CD-Proximicin-A system became electrophilic, enhancing its interaction with nucleophilic sites like DNA and RNA bases [64]. Protonating the imine polarizes the pi-electron cloud away from the reaction site. Electron density is pulled toward the positively charged nitrogen, suppressing Pauli repulsion with nucleophiles such as water [65]. Since the HOMO–LUMO gap also determines light emission wavelength, the pH-responsive system creates an optical cue indicating the dot’s presence in an acidic environment.

Moreover, Lopez-Munoz et al. designed and compared two types of chiral CDs covered in PLGA plastic coating: C6 (covered with oxygen-based functional groups) and C7 (covered in nitrogen-based groups). After three days in a neutral environment (pH 7.4), 57% of C6 was released while 42% of C7 was released. Yet, when the pH was lowered to 3, 56% of C7 was released in three days, compared to 49% of C6. Protonation of the nitrogen functional groups in C7 resulted in both increased hydrophilicity and electrostatic repulsion, improving water dispersibility [66]. These results are notable as CCDs have a distinct 3D orientation, giving them “lock-and-key” capabilities as biological receptors are also chiral. This gives them stereoselectivity: L-cysteine-capped (here meaning L-cysteine was used as a precursor) CCDs were able to specifically accumulate inside the Golgi apparatus via specific protein transporters. Normal CDs cannot leverage this pathway and would require charge or surface functionalization to attach to specific organelles. In this process, many CDs would likely be scattered across the cell or become trapped in endosomes or lysosomes [67].

#### 4.2.2. Redox-Responsive

Drug delivery systems in this category release cargo as a result of a change in electron transfer potential. Inactive cisplatin (Pt(IV)), a platinum-based chemotherapeutic drug, was attached covalently (amide bond, C=O) to carbon dots by Zhang et al. [68]. Afterwards, polyethylene glycol polymers (PEGs) were attached to remaining amine groups via a pH-cleavable benzoic imine bond, wrapping around the drug-dot system to form a protective shell. Once the nanoparticle system reaches the acidic tumor microenvironment (pH 6.8 compared to blood pH 7.4), protons attach to the nitrogen atom, creating a positive charge that makes the adjacent carbon more attractive to water, prompting hydrolysis. The imine is split into a benzaldehyde and an amine, and the PEG shell is shed [68,69].

Once the dot-drug system enters the tumor via endocytosis, glutathione (GSH), an overexpressed tripeptide in cancer cells, reduces the drug to its active form (Pt(II)). GSH’s free thiol group (-SH) performs a nucleophilic attack on the polar chlorido ligand on Pt(IV), forming a temporary intermediate bridge (Pt(IV)–Cl–S–GSH) through which GSH can donate electrons to the platinum atom’s d-orbitals. This prompts the shedding of two axial ligands on the drug’s top and bottom, putting cisplatin into its active form. As the bottom axial ligand formed the amide bond with the CD, the drug was also released [70]. This drug delivery example shows the versatility of CDs in two ways: redox- and pH-responsive mechanisms are not mutually exclusive but can work in tandem in a size-switchable hybrid platform.

### 4.3. Surface Functionalization

#### 4.3.1. Active Targeting

As mentioned in the third section, surface functionalization is a common strategy to promote active targeting. The surface of carbon dots is engineered with select ligands that bind to molecules that cancer cells commonly overexpress, promoting binding and recognition [71].

For instance, Dada et al. utilized folic acid’s natural attraction to folate receptors to deliver doxorubicin, an anticancer drug, to tumor cells. This is facilitated by the presence of amines on the CDs surface. After carbodiimide coupling, carboxylic acid (-COOH) groups on folic acid will form amide bonds with surface amines, thus binding folic acid to the CD exterior. Folic acid binding ultimately causes more precise drug release and structural integrity in transit, effectively treating folate-receptor-positive malignancies [72,73,74].

#### 4.3.2. Preventing Protein-Corona Formation

One obstacle that CD-based theranostic and drug delivery systems face is protein-corona formation. In the bloodstream, serum proteins like albumin and immunoglobulins coat the dot’s surface. This both promotes immune cell uptake and hides surface-functionalized ligands, thereby severely limiting therapeutic effects. Problematically, protein coronas also increase the size of CDs in vivo, limiting renal clearance and penetration capability [63,75,76].

Xie et al.’s GSH-CQDs’ surface amino acid groups formed hydrogen bonds with water molecules into a hexagonal arrangement, creating a rigid hydration shell that prevents protein adsorption and promotes colloidal stability [63]. Additionally, loading with doxorubicin did not disrupt this shell, demonstrating the compatibility of interactions.

Functionalization with zwitterions (molecules containing both positive and negative functional groups) has emerged as an alternative method to combat protein-corona formation. The mechanism is slightly different, but the end product is identical: zwitterions promote strong electrostatic interactions with water molecules, creating a hydration shell that leaves active targeting ligands intact [77].

**Table 1:** Usage of various types of CDs in drug delivery/theranostics.

CD and Doping	Surface Functionalization and/or Target Ligand	Receptor Target	Loaded Drug and Binding Type	Stimuli/Trigger	Key Mechanism and Outcome	Reference(s)
Unfunctionalized CD core	Imine (C=N) bond	N/A	Proximicin-A (PROXI), covalent link	Acidic pH (cancer cells)	pH-responsive drug release, protonation suppresses repulsion with nucleophiles, smaller bandgap	[64,65]
Chiral CDs (CCDs)	PLGA plastic shell, C6 (-O) vs. C7 (-N) groups	N/A	N/A	Acidic pH	pH-responsive CCD release in C7 due to protonation, “lock-and-key” capabilities	[66,67]
Amine-CDs	PEG shell (attached after drug)	N/A	Pt(IV) prodrug, amide bond	Both acidic pH and glutathione (GSH)	PEG shed at acidic pH; GSH reduces Pt(IV) to active Pt(II)	[68,69]
Folic acid-CDs (FA-CDs)	Folic acid	Folate receptors	Dox, both covalent and noncovalent bonds tested	Receptor-mediated uptake	FA successfully targeted overexpressed folate receptors, precise drug release	[72,73,74]
Glutathione CQDs (GSH-CQDs)	Amino acid groups, rigid hydration shell	N/A	Dox, pi–pi stacking	N/A	Hydration shell prevented protein adsorption and promoted colloidal stability	[63]
Red-emitting CDs (RCDs)	N/A	N/A	N/A (photosensitizer core)	Light activation	Core size dictates type-I PDT; surface states dictate type-II PDT	[78]
Lead sulfide core quantum dots (QDs)	Cadmium sulfide (CdS) and zinc sulfide (ZnS) shell	N/A	N/A (methylene blue, PET quencher)	Matrix metalloproteinase	MMP cleavage results in NIR-II wave emission. Free MB could drive ROS production if activated by light	[28]

N/A: Not Available.

### 4.4. Photodynamic Therapy (PDT)

The same quantum mechanism that causes organic dyes to photobleach (singlet state transitioning to a triplet state via ISC [37]) makes the carbon dot a potentially effective cancer therapy. In PDT, photosensitizers facilitate the creation of ROS that then destroy target malignant cells from within [78]. Heteroatom doping ensures that CDs readily populate the triplet state by accelerating ISC, overcoming the sp^2^ carbon lattice’s rapid exciton dissipation [42]. As these doping parameters can be synthetically modified, CDs represent versatile photosensitizers in PDT, making them ideal for personalized, case-specific cancer treatment.

Moreover, CDs are able to circumvent the hypoxic tumor microenvironment that makes type-II PDT ineffective. Zhang et al. prepared red-emitting CDs that were able to participate in type-I PDT. In this mode of PDT, when excited, the photosensitizer transfers electrons to surrounding substrates to yield superoxide anions without relying on the presence of oxygen. In addition, both type I and type II ROS formation depended on the carbon dot’s core diameter and surface states, respectively, demonstrating the clinical potential of applying an understanding of quantum mechanisms to theranostics [78].

Interestingly, in the aforementioned study by Jeong et al., methylene blue was attached to act as a photoexcited electron transfer (PET) quencher. When the dot encounters matrix metalloprotease, which is overexpressed in many cancers, methylene blue is cut free from the dot, allowing the electrons to recombine with their holes and emit NIR-II waves. Decoupled methylene blue could also be used in PDT, as it produces ROS when exposed to light of 630–680 nm. The reactive ROS also breaks methylene blue’s chemical structure, ensuring the toxic substance does not stay active in vivo. The study used heavy metal-containing quantum dots, so a biocompatible CD-based alternative is necessary. Yet, this highlights the creative, innovative strategies that drive the development of new theranostic methods [28,79]. The various structural variables in CDs discussed in Section 2, Section 3 and Section 4 are linked to their effects in Table 2.

## 5. Bottlenecks and Future Implications

### 5.1. Renal Clearance and Pharmacokinetic Profiling

Smaller nanomaterials are intriguing as a modern mode of treatment because of their rapid renal clearance. Studies estimate that CDs can be at most about 5–6 nm in diameter for glomerular filtration in the kidneys. This is precisely why preventing protein-corona formation is an essential part of CD-based theranostics: adsorption of serum proteins can increase CD diameter by over 15 nm, preventing renal excretion [77,80]. While some larger nanoparticles (above 8 nm) have been able to escape into urine, this mechanism is not understood well enough to engineer larger, easily clearable CDs [81]. Other factors such as shape, charge, and rigidity affect this threshold. First, nanoparticles with a dimension greater than zero (e.g., 1D rods and 2D sheets) can be longer than 100 nm and still bypass the renal barrier, as shape influences both behavior and orientation under blood flow and interaction with the glomerular filtration barrier. Next, in the 6–8 nm range, positively charged nanoparticles are generally cleared faster than neutral and negatively charged molecules, in part due to the negatively charged glomerular basement membrane, one of the three critical layers of the glomerular filtration barrier. Finally, the threshold for diameter increases for deformable nanomaterials. While the exact number is still uncertain, 400 nm long tubes and 60 × 90 nm rectangles have successfully undergone renal clearance in mice [82]. Despite this advantage, while designing CD-based theranostic systems, it is important to consider that decreased rigidity can have unfavorable effects like premature, unintended drug leakage, especially in the extracellular matrix.

Environmental concerns must be considered as well. Without nanofiltration membranes, wastewater systems cannot guarantee the complete removal of nanoparticles, potentially an ecotoxicological risk, as the effects of carbon dots on marine life and ecosystems are largely unknown [83].

Renal clearance is crucial before clinical use of CDs, as it helps bypass long-term organ toxicity. Another troublesome effect of protein-corona formation is the promotion of immune cell uptake. Specialized macrophage cells like Kupffer cells in the liver and red pulp macrophages in the spleen recognize opsonins on the corona, causing accumulation in these areas [63,84]. Even though histopathological inspections have portrayed CDs as non-toxic and biocompatible, H NMR metabolic approaches reveal that in mice, the sequestration of CDs triggers immune responses (decreased amounts of five essential amino acids in the spleen) and disrupts both cell membrane functions (blockage of choline transporters and insulin-receptor binding) and liver metabolic pathways (impaired clearance of lactate and significant increase in glutamine levels) [85]. The CDs used in this study do not appear to be doped or surface-functionalized [86]. This evidence not only reveals that toxic effects cannot always be attributed to synthetically engineered changes, but also that before regulatory approval, tests involving CDs must go through advanced metabolic tests.

### 5.2. Manufacturing Bottlenecks

Despite extensive in vitro and in vivo research demonstrating theranostic potential, several obstacles stand before the clinical application of CDs.

One of the nanomaterial’s main challenges is structural heterogeneity. This stems from a variety of causes. Firstly, since new CD recipes are created by scientists all over the world, precursor properties, especially in biomass-derived materials, vary significantly. Moreover, CD function is also determined by a plethora of factors, including, but not limited to, synthesis method, surface functionalization, heteroatom doping, and core diameter [87]. Due to the complexity of reproducing CDs, even the slightest difference results in structural inconsistency, which can affect photoluminescence, drug loading, and other key theranostic components. Consequently, several structural and electronic properties are disrupted, such as the exact location of a surface state and sp^2^/sp^3^ character. This makes large-scale yet controlled synthesis of CDs difficult, an obstacle when reproducibility is necessary for any theranostic system. Additionally, heteroatom doping, surface functionalization, and the specific precursor used all must be standardized before reproducible theranostic performance.

Regulatory ambiguity also hampers progress toward approval of CD-based therapies. Despite substantial successes in nanotechnology like the COVID-19 mRNA vaccine, the FDA currently lacks specific guidelines and Good Manufacturing Practice protocols for nanomaterials, instead applying current regulations for standard pharmaceutical drugs or medical devices [88]. To date, this is still a flawed approach, as these therapies are evaluated on their chemical composition, while nanomaterials are largely defined by surface properties, physical geometry, and size. Future laws could take inspiration from programs like the US NCI-NCL or the EU-NCL, which provide more robust guidelines and methodologies for utilizing nanomaterials in health products [89].

To simplify mass production by avoiding the need for drug conjugation, more methods that prepare CDs with intrinsic therapeutic properties can be developed. For example, Wang et al. used ginseng to synthesize CDs that inhibited growth of select cancer cells without harming healthy cells at lower concentrations [90]. However, these CDs are limited by the lack of active-targeting ligands, making precise therapy more difficult and protein-corona formation more likely. Consequently, surface functionalization strategies could be viewed as a strategy to personalize and optimize the efficacy of future carbon-dot-based therapies. Finally, artificial intelligence and machine learning algorithms could also be used to both predict CD synthetic outcomes and properties, in turn aiding batch-to-batch heterogeneity [91].

## Figures and Tables

**Table 2:** Summary of CD structural variables and their effects.

Structural Variable	Electronic Structure	Excited-State Processes	Photophysical Outputs	Theranostic Functions	Translational Performance	Reference(s)
Sp^2^ domain size	Larger: increase pi-electron delocalization and reduce HOMO–LUMO gap Smaller: strengthen quantum confinement effect	Non-radiative relaxation, radiative recombination, exciton confinement	Tunable emission wavelength, increased two-photon absorption in larger domains	NIR emission, up-conversion fluorescence	Deep-tissue imaging with increased signal-to-noise ratio	[19,20,21,33,50,51]
Sp^2^/sp^3^ character	Sp^2^ clusters provide delocalized pi-states; sp^3^ domains serve as insulators	Photoluminescence, quantum confinement	sp^3^-hybridized carbons create surface states	Both used to tune HOMO–LUMO gap	Synthetic route to controlling optical behavior, but reproducibility is still a problem	[11,19,50,51,52,53]
Surface states	Localized energy levels created by sp^3^-hybridized carbons, heteroatom doping, surface functional groups, etc.	Excited electrons relax at surface states before non-radiative or radiative recombination	Tunable emission wavelength	Can be engineered to tune HOMO–LUMO gap to facilitate NIR imaging	Heterogeneity is a major obstacle to consistent optical performance	[22,23,24,25,30,44]
Heteroatom doping	Modifies surface charge, creates surface states, etc.	Accelerated intersystem crossing; heavy-atom effect increases spin–orbit coupling	Tunable emission wavelength (can work in tandem with extended sp^2^ domain); increased quantum yield	Can be used to tune both emission wavelength and ISC kinetics (PDT)	In situ doping can be leveraged for active targeting, but excessive doping can introduce non-radiative defects and decrease fluorescence	[30,42,52,53,54,55,56,78]
Surface functionalization	Functional groups and attached ligands to selectively bind to molecules	N/A	N/A	Active targeting, smart drug release, prevention of protein-corona formation	Improves targeting and pharmacokinetic behavior, but can complicate reproducibility	[57,58,63,64,65,66,67,68,69,70,71,72,73,74,75,76,77]

N/A: Not Available.

## Data Availability

No new data were created or analyzed in this study. Data sharing is not applicable.
